# Obituary Notice of Arago

**Published:** 1854-02

**Authors:** 


Obituary Notice of Arago.—A life of so much labor had worn down his health. Although attacked with diabetes, he still contemplated putting the last touch to his unfinished works. Bright's malady set in and aggravated his situation, which was complicated with dropsy of the abdomen, attended with effusions, and swelling of the extremities. All announced his approaching end; yet his mind was not for a moment obscured. Shortly before his death, although blind, he superintended in some difficult researches; he asked M. Babinet to prepare for him a table of more accurately determined numbers for the lengths of undulations, that he might bring to completion a memoir on interferences; and he finished the editing of his Physical researches on the Planets, &c.,

&c. He died in the midst of these arduous occupations, on the 2nd of October, at the age of sixty-seven years and a half, a few minutes after having shaken the hand of M. Biot.

We have mentioned some of the works which Arago accomplished in his younger days. These works were completely eclipsed by the discoveries to which his name has since become attached, which embrace the following principles:

1. The discovery of chromatic and rotatory polarization.
2. That of Electro-magnets.
3. That of the magnetism which is developed when bodies are revolved near a magnet.

Arago was an Encyclopedic genius. Science, Literature, Political and Social Economy, his vast intelligence embraced all with equal ability. His powerful faculty of assimilation, popularization, and of application of principles, placed him everywhere in the first rank. Whether Orator or Professor, he shone with brilliancy both in political and scientific assemblages. He was distinguished for the perspicuity and elegance of his style, and occupies an eminent place among the prose writers of France.

In the midst of so much grandeur, Arago led a most modest life. He considered as lazy whoever did not work fourteen hours a day; and such days were for him days of repose. Although so absorbed with his occupations, he still found time to appear in the society of Paris as one of its most spirited conversationists.

While devoted to continued labor, he completely forgot his own interests, and had only what was barely necessary for the support of his family.—*American Journal of Science.*

